# Interface Chirality: From Biological Effects to Biomedical Applications

**DOI:** 10.3390/molecules28155629

**Published:** 2023-07-25

**Authors:** Liting Guo, Yanqiu Guo, Rui Wang, Jie Feng, Nannan Shao, Xiaolin Zhou, Yunlong Zhou

**Affiliations:** 1 Joint Research Centre on Medicine, Affiliated Xiangshan Hospital, Wenzhou Medical University, Ningbo 315700, China; 2 Zhejiang Engineering Research Center for Tissue Repair Materials, Wenzhou Institute, University of Chinese Academy of Sciences, Wenzhou 325000, China; 3 School of Pharmacy, Queens University Belfast, 97 Lisburn Road, Belfast BT9 7BL, UK

**Keywords:** chirality, interface, cell fate, tissue repair, immunity, application

## Abstract

Chiral surface is a critical mediator that significantly impacts interaction with biological systems on regulating cell behavior. To better understand how the properties of interfacial Chirality affect cell behavior and address the limitations of chiral materials for biomedical applications, in this review, we mainly focus on the recent developments of chiral bio-interfaces for the controllable and accurate guidance of chiral biomedical phenomena. In particular, we will discuss how cells or organisms sense and respond to the chiral stimulus, as well as the chirality mediating cell fate, tissue repair, and organism immune response will be reviewed. In addition, the biological applications of chirality, such as drug delivery, antibacterial, antivirus and antitumor activities, and biological signal detection, will also be reviewed. Finally, the challenges of chiral bio-interfaces for controlling biological response and the further application of interface chirality materials for biomedical will be discussed.

## 1. Introduction

The interface is one of the most crucial factors for the interaction of life subunits and physical externalities because the multi-level interaction between living systems and environments usually occurs at their interfaces [1,2,3]. Chirality is a natural phenomenon that is general and crucial in various organisms and substances [4]. In recent years, many studies have shown that chiral interface recognition occurs at the different dimension-interfaces of some cell membranes, cells, tissues, or organs in organisms, which can control various biological processes [5]. In order to study the mechanism of chiral biological phenomena in nature and develop new biomedical materials, it is necessary to understand the interaction between cells or organisms and different types of interfaces. Therefore, studying the biological effect of chiral interfaces is meaningful and crucial.

Exploring the construction of chiral interfaces and their impact on various biological effects, especially cell fate, tissue repair, and immune response, is an effective and necessary way to understand natural chirality and further develop its applications [6,7,8,9]. There have been many studies reported to prepare various types of chiral interfaces and to study their effects on cells, tissues or organisms, which greatly accelerate the chiral mechanism and promote the application of chiral effects in biology, medicine, and other related fields [10,11]. Furthermore, the understanding of chiral interface materials helps to promote chiral applications in diagnosis and treatment. So far, many studies have applied the biological effects triggered by chiral interfaces to fields such as drug delivery, antibacterial, antivirus and antitumor activities, and biological signal detection [12,13,14]. These studies help us better understand how chiral interface properties affect the behavior of cells or living organisms and how to better achieve biomedical applications of chiral interfaces.

**Figure 1:**
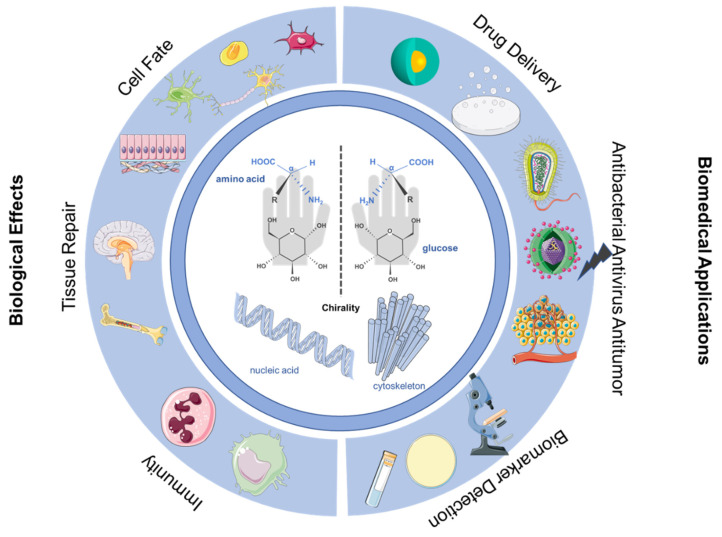
Schematic diagram of biological effects and biomedical applications.

In this review, we mainly focus on the new phenomena and recent developments in chiral bio-interfaces. In particular, the interface chirality mediating cell fate, tissue repair, and organism immune response and the biological applications of interface chirality, such as drug delivery, antibacterial, antivirus and antitumor activities, and biological signal detection, will be reviewed (Figure 1). Ulteriorly, the challenges and application of chiral bio-interfaces for biomedical will be discussed.

## 2. Chiral Interface for Controlling the Cell Fate

The development of biomaterials prompts us to study the interaction between intrinsic chiral bio-systems and artificial chiral materials. The chirality of material has been demonstrated to be an important mediator for regulating cell fate, in which the chirality can influence biological processes such as protein adsorption, cell adhesion, and consecutive behaviors. Indeed, nature itself has already evolved to chiral preference. And chirality is inherent in molecular recognition operating within biological systems. For example, cell membranes are formed by chiral amphiphiles, collagen is formed from chiral helical fibers, and the widely cited double helix in genetic material is also chiral. In addition, cell chirality is also present, which can be regulated by actin-binding proteins such as α-actinin and can also be mediated by certain signaling pathways [11,15]. Recently, research suggested that the chiral moieties present on the material’s surface have a great impact on their interaction with biological systems [16,17,18,19]. Despite all these above-mentioned backgrounds, few review articles have specifically addressed the effect of chirality in guiding cell fate [10,20]. In this review, we mainly focus on the development of chiral bio-interfaces for the controllable and accurate guidance of cell fate. In particular, we will discuss how cells themselves sense chirality and respond under chiral stimulus.

Up to now, much research has been conducted to study the interaction between intrinsic chiral biosystems (proteins, nucleic acids and cells) and chiral-based materials. There is a reasonable conjecture that diastereomeric energy differences lead to differences in biological effects. The interactions between biological systems and chiral surfaces typically exhibit a diastereomeric nature between enantiomeric guest molecules and homochiral biological receptors. This characteristic leads to potential diastereomeric energy differences between homochiral vs. heterochiral interactions, L-L vs. L-D for instance [21,22,23]. Such research not only helps us understand chiral response mechanisms but also promotes the applications of the chiral effects in biological systems. Many chiral-based bio-interfaces have been constructed, and these interfaces with a series of unique properties brought by the intimate interactions between cells and chiral interface, which make chiral interfaces to be a powerful platform to guide cell fate in a more controllable and accurate way, such as the control of cell adhesion, cell proliferation, migration, and stem-cell differentiation.

### 2.1. Chiral Effects on Cell Toxicity or Viability

Cell toxicity or viability are the most obvious responses of cells to the stimulation of the chiral interface. It has been found that opposite enantiomers in the case of chiral structures can interact quite differently with biological systems. A clear understanding of chirality-mediated cytotoxicity will help us to develop much safer chiral medicine.

Nie’s team prepared glutathione (GSH) modified CdTe Quantum Dots (QDs) and found that L-GSH-QDs showed greater cytotoxicity in human hepatoma HepG2 cells [24]. They further revealed that the induction of autophagy was chirality-dependent progress, with L-GSH-QDs inducing a higher level of autophagy. This work points out a new way for the regulation of cellular activities by the use of chiral bio-interface material. Similarly, Kotov’s team adopted chiral amino acids-cysteine to prepare chiral graphene QDs (GQDs) and found that L-GQDs showed slightly higher overall biocompatibility in HepG2 cells. In addition, they revealed that D-GQDs tended to accumulate more within the cellular membrane than L-GQDs by molecular dynamics simulations [25].

Gao et al. adopted poly-acryloyl-L/D-valine (L/D-PAV) chiral molecules to modify the surfaces of Au nanocubes and Au nanooctahedras [26]. They found that the cell viability exposed to the PAV-capped Au nanoparticles (NPs) was chirality-dependent. The L-PAV-capped Au nanocubes and Au nanooctahedras exhibited larger cytotoxicity than the D-PAV-coated ones, respectively. They further indicated that such kind of difference in cytotoxicity was positively correlated with the cellular uptake amount and thereby the production of intracellular reactive oxygen species (ROS).

**Figure 2:**
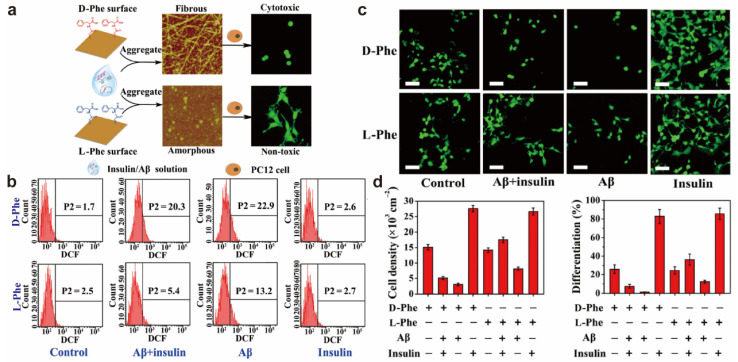
(**a**) The morphology and cellular responses of crossaggregates of Aβ and insulin on D- and L-Phe surfaces. (**b**) Dichlorofluorescein diacetate (DCFH-DA) was used as the probe to analyze intracellular ROS. (**c**) Fluorescence microscopy imaging of PC12 cells. The scale bar is 40 μm. (**d**) Estimation of cell density and statistics for the differentiation rate of PC12 cells [27].

Qu’s team studied the effect of phenylalanine-modified chiral surfaces on the cross-fibrosis of insulin and Aβ, and cellular responses [27]. On the D-Phe-modified surface, Aβ induces insulin to co-aggregate into β-rich fibers and cross fibers that lose biological activity, significantly increasing intracellular ROS levels and showing significant cytotoxicity, instead on the surface of L-Phe. This result shows that surface chirality can affect the cross-fibrosis of Aβ and insulin and the cytotoxicity of their aggregates (Figure 2).

**Figure 3:**
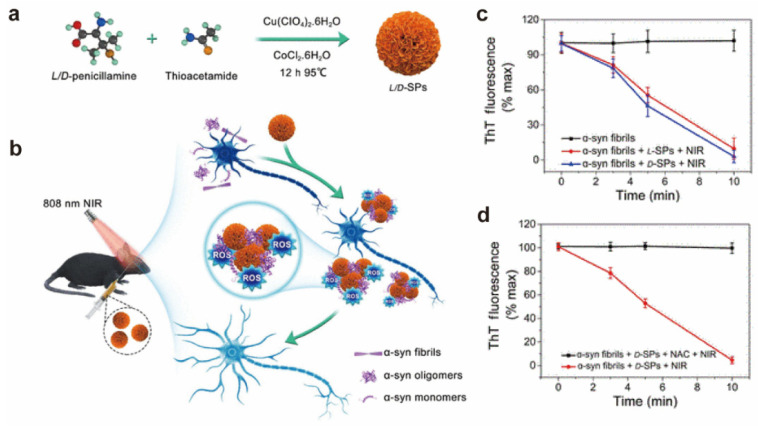
(**a**) Schematic of L/D-Pen-modified L/D-Cu_x_Co_y_S SPs. (**b**) Illustration of the inhibition and disassembly effects of D-SPs on α-syn aggregation and mitigation of potential neurotoxicity in a PD mice model. ThT fluorescence monitors the depolymerization kinetics of α-syn fibrillization after (**c**) incubation with or without L/D-SPs in the same reaction time and (**d**) with or without ROS inhibitor using aliquots of the reaction [28].

Kuang’s team synthesized chiral Cu_x_Co_y_S inorganic ultraparticles (L/D-SP) with L- or D-penicillamine (L/D-Pen) as the ligand and found D-SP has a higher α-synthetic fiber depolymerization capability than L-SP under near-infrared light [28]. Moreover, near-infrared illumination activates D-SP to produce a large amount of reactive oxygen species, destroying the stability of the α-fibrils β-sheet conformation, preventing fibrillation of monomers, and reducing their cytotoxicity (Figure 3). This study paves the way for the use of cell toxicity response to chiral inorganic ultra particles to treat diseases.

Similarly, Wu et al. studied the effect of L/D-Pe-Au NP on cells by forming a stable Pe-Au NP [29]. It was found that L-Pe-Au NPs produced potent cytotoxicity to cells at different concentrations, while D-Pe-Au NP was much less toxic than L-Pe-Au NP. L-Pe-Au NPs cannot be used directly as a drug inhibitor in the treatment of Alzheimer’s disease. This study reveals the significance of chiral nanoparticles in neurotoxicity too.

### 2.2. Chiral Effects on Cell Adhesion

Cell adhesion is one of the fundamental cell activities, which is strongly regulated by materials’ chirality. When the chiral materials come in contact with biological systems, they immediately interact with each other through a kind of physics-bio interface, and protein adsorption on such interface usually first occurs. Previous studies have already demonstrated that protein adsorption at interfaces is closely dependent on the chirality of materials [30,31,32,33,34]. The chirality-induced protein adsorption difference might significantly influence signal transduction and cell response, which in turn serve as a regulator for mediating selective cell adhesion.

Sun et al. adopted enantiomers of N-isobutyryl- L/D-cysteine (L/D-NIBC) to construct chiral self-assembled monolayers (SAMs) and found that the quantity of adhered cells on the L surface was higher than that of the D surface and the cells on the L surface more tended to deform and spread along the surface than D surface [35,36]. They further investigated the stereoselective cell behavior on a pair of chiral polymer brushes consisting of the chiral acryloyl derivatives of L/D-valine (L/D-Val). It was founded that the cells can adhere, grow, spread, and assemble much better on the L-amino acid-based polymer film than on the corresponding D film. These works indicate that chiral surfaces could be used to modulate cellular behavior.

**Figure 4:**
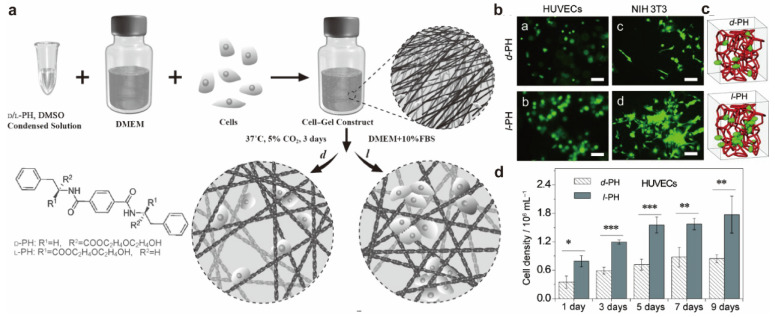
(**a**) The different cell-adhesion and cell-proliferation behavior in the enantiomeric nanofibrous hydrogels. (**b**) Fluorescence microscopy images of HUVECs and NIH 3T3 cells in D-PH and L-PH hydrogels. Scale bar: 50 μm. (**c**) Schematic representation of different cell adhesion behaviors in the enantiomeric nanofibrous hydrogels. (**d**) Quantitative data for HUVECs cultured in D-PH and L-PH hydrogels. * *p* ≤ 0.05, ** *p* ≤ 0.01, *** *p* ≤ 0.001 [6].

Three-dimensional topographical chirality is an important part of cell microenvironments. Most materials, signals, and energy exchanges between cells and their microenvironments occur at three-dimensional nanostructured cell interfaces. The cell adhesion behavior could also be affected by the chirality of three-dimensional (3D) hydrogel [6,37]. Feng et al. employed the two enantiomers of a 1,4-benzenedicarboxamide phenylalanine derivative as supramolecular gelators (D-PH and L-PH) as supramolecular gelators and investigated the influence of the chirality on cell adhesion and proliferation. They found that left-handed helical nanofibers can increase cell adhesion and proliferation (Figure 4).

Shefi’s team fabricated chiral surfaces by assembling natural chiral amino acids-cysteine of opposite configurations (D-and L-) onto gold surfaces and investigate the effect of chirality on cell behavior. And they found that exposure to the L-cysteine enantiomer inhibited neuronal attachment more severely than exposure to the D-cysteine enantiomer. This work indicates that chirality could act as regulator of neuronal cell behavior [37].

Subsequently, Kuang’s team modified single-layer plasmonic chiral gold NPs membranes with L-or D-penicillamine to interact with cells and collect cells noninvasively [38]. After culture, the cells on the L-PEN-NP film were more than the D-PEN-NP film. The cell shedding rate of L-PEN-NP film exposed to left circularly polarized light (LCP) was higher than the D-PEN-NP film.

### 2.3. Chiral Effects on Stem Cell Differentiation

Stem cells have the ability of self-renewal and differentiation into specialized cell types, these unique properties led to broad applications in tissue engineering and regenerative medicine for treating several diseases, including neurological, hepatic, hematopoietic and diabetic diseases. Stem cell behavior is thought to be regulated by the microenvironment. In order to develop stem cell-based applications, it is essential to understand the response of stem cells to chiral materials.

Ji’s team self-assembled L/D-phenylalanine-derived molecules (LP and DP) into left-handed and right-handed helical fiber networks, respectively [39]. They found that DP nanofiber membrane enhanced retinal progenitor cell proliferation by activating Akt and ERK pathways, while LP chiral nanofiber membrane-maintained stem/progenitor cell phenotype by promoting vimentin expression. This work indicates that chiral surfaces could act as indirect regulators of nerve stem cells and provide a reference for studying chiral materials in the future.

Yoshimoto et al. explore the effect of chirality on stem cell fate based on Human bone marrow mesenchymal stem cells encapsulated by a self-assembled L-and D-type ultra-short peptide Fmoc-Phe-Phe soft fiber hydrogel system [40]. Interestingly, the increment rate of MSC in L-Gel was higher than D-Gel, and the cells cultured in D-Gel remained highly undifferentiated for a month. The expansion of MSCs in an undifferentiated state is essential for the success of cell therapy, so the D-type peptide material can be used as a scaffold for the regenerative medicine of stem cells [41].

To explore the chiral effects of molecules and supramolecules in nanofibers, Feng’s team synthesized L/D-phenylalanine-based self-assembled nanofibers [42]. The results showed that compared with molecular chirality, supramolecular spiral chirality amplifies the diffusion and proliferation of cells. This broadens the scope of research for the application of chiral biomaterials in biology and medicine. Recently, their team continued to explore the effects of the enantiomer L/D-phenylalanine gel factor (LPG, DPG), respectively, and their racemic mixture (RPG) on the differentiation of retinal progenitor cells [7]. Compared with left-handed LPG nanofibers and racemic RPG nanofibers, dextron-DPG nanofibers significantly promote neuronal differentiation, migration and synaptic formation of RPC. Further analysis found that this difference may be due to the activation of the retinoic acid (RA) metabolic pathway due to increased adhesion of RBP4 on DPG nanofibers, which can trigger the differentiation of stem/progenitor cells. This work provided new ideas for the treatment of neurodegenerative diseases. And, Feng’s team reported that the chirality of a constructed 3D extracellular matrix (ECM) differentiates mesenchymal stem cells from diverse lineages of osteogenic and adipogenic cells by providing primary heterogeneity [43].

**Figure 5:**
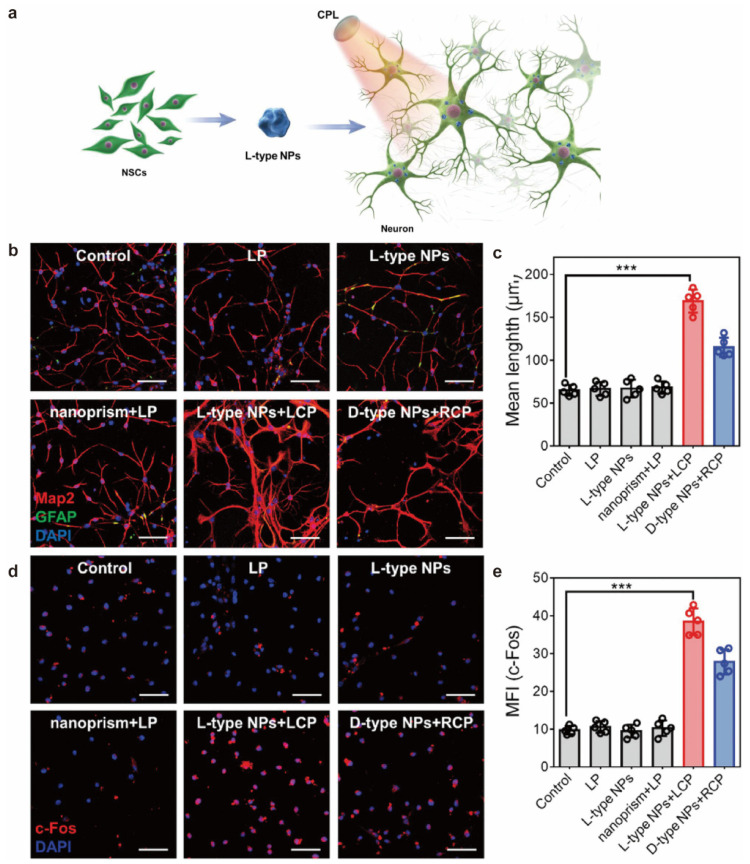
(**a**) The different cell-adhesion and cell-proliferation behavior in the enantiomeric nanofibrous hydrogels. (**b**) Confocal images of NSCs under different experimental conditions. (**c**) Mean lengths of neurites in differentiated NSCs. (**d**) Confocal images of c-Fos protein in NSCs incubated with different experimental conditions. (**e**) Mean fluorescence intensity (c-Fos). *** *p* ≤ 0.001. Scale bar, 100 μm [8].

Both molecular chiral and nanofiber structural chirality have been shown to regulate cellular behavior and influence cell differentiation. In addition, nanoparticles have become new targets for chiral effect research due to their surface effect and amplification effect. Zang et al. synthesized an AuNP-loaded L/D-AuNC-based nanomaterial that was formed and used for the differentiation study of MSCs [44]. MSCs on D membranes have higher cell density and spread area than L membranes. After differentiation induction, more MSCs on the L membrane differentiate into lipoblast and more differentiate into osteoblasts on the D membrane. At the same time, Kuang‘s team studied the effect of chiral NPs with strong chirality under the irradiation of circularly polarized light on the differentiation of neural stem cells in mice (Figure 5) [8]. The results showed that chiral NP promotes neural stem cell differentiation under CPL light, and L-type NP is significantly better than D-type NP. The promotion of neural stem cell differentiation by such strong chiral materials provides an opportunity for the treatment and regeneration of nerve diseases.

In addition, the interaction of the chiral-shaped micro-structured ECMs to cellular chiral alignments and differentiations also be considered as an important subject. Kim et al. prepared twisted wrinkle-shaped chiral micropatterns using biaxial and asymmetric flexion methods and cultured myoblasts on each of the two enantiomeric chiral micropatterns in mirror reflection shape. The results suggest that myogenic cells exhibit enantioselective recognition of the structural chiral microenvironment, which can promote cell alignment and differentiation [45]. This research contributed to the understanding of enantioselective interactions between cell chirality and the extracellular environment.

## 3. Chiral Effects on Tissue Repair

The stem cell therapy has become a powerful method of tissue repair and regeneration. Taking nerves as an example, the role of neural stem cells in Neurotherapy is mainly to promote the differentiation of neurons and oligodendrocytes. Due to chiral-specific interactions in organisms, chiral structures, as the basic structural units of organisms, are important for regulating cellular behavior. By regulating the chirality of the interface, the differentiation direction of stem cells can be controlled, further tissue repair can be promoted.

Nanomaterials with a variety of enzymatic activities have attracted widespread attention. Kuang’s team prepared uniform and porous Cu_x_O NCs with L-phenylalanine (L-Phe) as the structural guide [46]. In the PD mice model, L-Cu_x_O NC can significantly promote dopamine synthesis and thus saves memory loss in Parkinson’s disease (PD) model mice.

**Figure 6:**
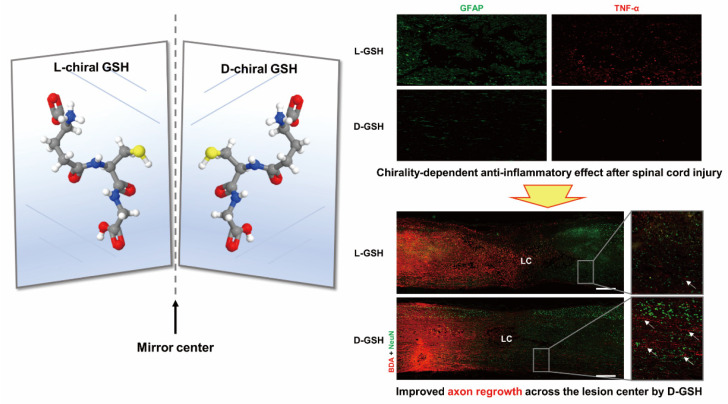
Neuroinflammation forms a glial scar following a spinal cord injury (SCI). The injured axon cannot regenerate across the scar, suggesting permanent paraplegia. Molecular chirality can show an entirely different bio-function by means of chiral-specific interaction. It was reported that D-GSH suppresses the inflammatory response after SCI and leads to axon regeneration of the injured spinal cord to a greater extent than L-GSH. Scale bar, 500 μm [47].

Sohn’s team investigated the chiral properties of glutathione in anti-inflammatory effects in a spinal cord injury model (Figure 6) [47]. Nerve damage induces an excessive local inflammatory response that leads to glial scarring, which can lead to permanent damage. Research on GSH in vivo showed the L-chiral form of glutathione (L-GSH) has anti-inflammatory effects.

Feng et al. use the enantiomer L/D-phenylalanine gel factor (LPG, DPG) and its racemic mixture (RPG) to mimic the chiral extracellular microenvironment [7]. It was shown that the stereo affinity of DPG for RBP4 was higher than that of LPG, which eventually led to enhanced neural differentiation of RPCs on DPG. This achievement has far-reaching significance for the treatment of neurodegenerative diseases by using the chiral structure of the extracellular microenvironment to control RPC differentiation.

Kuang group found that chiral nanoparticles (NPs) with strong chirality can effectively accelerate the differentiation of mouse neural stem cells (NSCs) to neurons under near-infrared light, with L-type NPs being higher than D-type NPs [8]. In addition, under the synergistic action of near-infrared radiation, the clearance of L-type NPs to amyloid protein and phosphorylated p-tau protein is higher than D-NPs, respectively. This exploration provides an opportunity for the application of chiral materials in the treatment of neurodegenerative diseases. In addition, the Kuang group prepared chiral L/D-Cu_x_Co_y_S ultrafine particles L/D-SP [28]. Notably, L-SP under near-infrared irradiation can effectively break down alphasyn aggregates. These findings provide ideas for the use of chiral SP in the treatment of neurodegenerative diseases under near-infrared light.

## 4. Chiral Effects on Immunity

Immunity usually refers to the body’s ability to resist the invasion of pathogenic microorganisms and resist various diseases. The immune defense and immune monitoring functions are closely related to recognition ability, and chiral interfaces play an important role in immune recognition.

Jeong and his colleagues prepared poly(ethylene glycol)-poly(L-alanine-co-L-phenyl alanine) (PEG-L-PAF) and poly-(ethylene glycol)-poly(D-alanine-co-D-phenyl alanine) (PEG-D-PAF) and studied the effect of chirality on acute inflammation [48]. They found that only PEG-L-PAF could be significantly degraded by cathepsin B and elastase. In addition, PEG-D-PAF gel could cause more acute inflammation compared to PEG-L-PAF gel. This work pointed out the significant difference in chirality in vivo experiments in acute inflammation.

**Figure 7:**
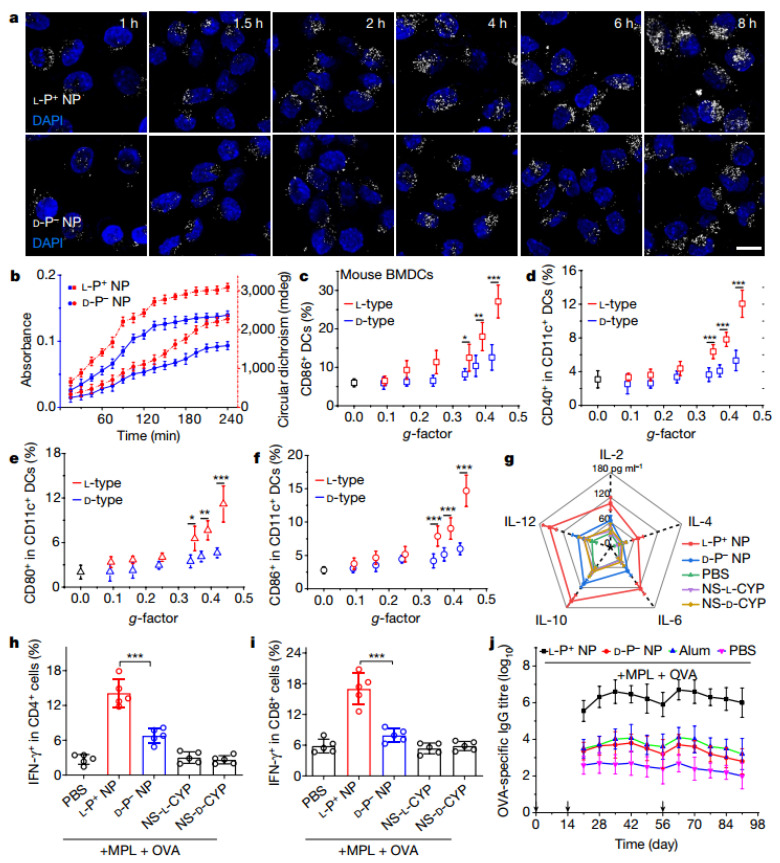
(**a**) Two-photon luminescence imaging of mouse BMDCs incubated with L-P^+^ or L-P NPs. Scale bars, 10 μm. (**b**) Maximum absorption (blue) and the sum of absolute values for two circular dichroism extrema (red) varied with incubation time for mouse BMDCs incubated with L-P^+^ NPs or D-P^−^ NPs. (**c**) CD86 levels were measured by flow cytometry. Expression of CD40 (**d**), CD80 (**e**) and CD86 (**f**) in recruited CD11c^+^ DCs. (**g**) Expression of IL-2, IL-4, IL-6, IL-10 and IL-12 in serum measured by ELISA. IFN-γ^+^ CD4^+^ (**h**) and IFN-γ^+^ CD8^+^ (**i**) T cells in spleens measured by flow cytometry. (**j**) Anti-OVA antibody titres in mouse serum. * *p* ≤ 0.05, ** *p* ≤ 0.01, *** *p* ≤ 0.001 [9].

Xu’s team find that achiral and left- and right-handed gold biomimetic nanoparticles show different in vitro and in vivo immune responses. They find that nanoscale chirality can regulate immune cell maturation in vivo and in vitro. Left-handed nanoparticles show substantially higher efficiency compared with their right-handed counterparts as adjuvants for vaccination against the H9N2 influenza virus, opening a path to the use of nanoscale chirality in immunology (Figure 7) [9].

**Figure 8:**
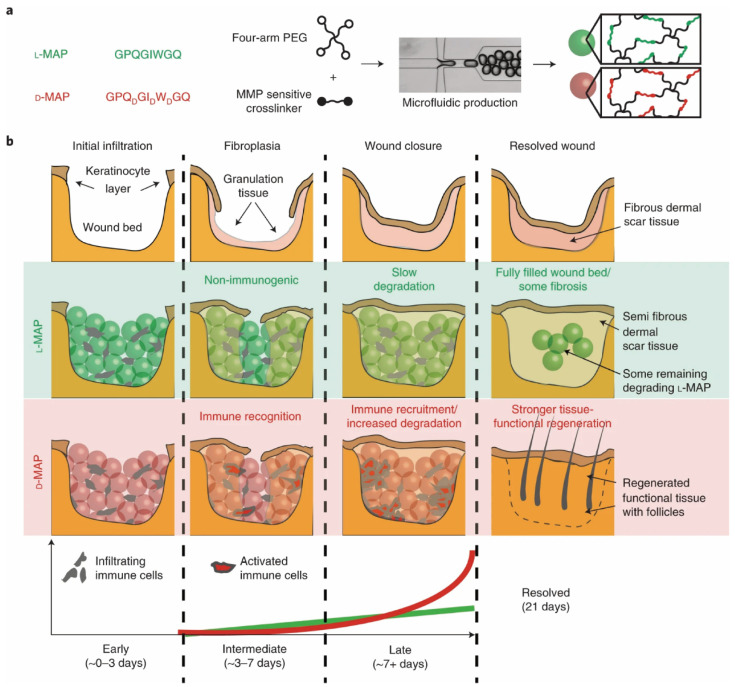
(**a**) Representation of the MMP cleavage sequences, amino acid chirality within the crosslinking peptides and microfluidic formation of the hydrogel microbeads that incorporate L-or D-chirality peptides. (**b**) The use of L- or D-MAP in a wound-healing model demonstrates that both the L-MAP (green) and D-MAP (red) hydrogels fill the wound defect [49].

Griffin et al. have studied the effect of chirality switching of peptides to promote the growth of tissue. The persistence of D-hydrogel at the wound site is minimal compared to the L-hydrogel and the D-hydrogel exhibits the wound-induced hair neogenesis. The L-microporous annealed particle (MAP) had more accumulation of CD11b cells, while the D-MAP and 1:1 L/D-MAP exhibited strong accumulation of CD11 expressing myeloid cells at the wound site. The results showed that D-MAP is involved in the enhanced immune response and degradation by immune cells (Figure 8) [49].

## 5. Application of Chiral Interface

Chiral materials play indispensable roles in maintaining numerous physiological processes, such as signaling, site-specific catalysis, transport, protection, and synthesis. Chiral materials usually exhibit extraordinary properties, such as high g-factor values, broad distribution range, and symmetrical mirror configurations. Because of these unique characteristics, there is great potential for application in the fields of biosensing, drug delivery, early diagnosis, bio-imaging, and disease therapy [50]. With the development of chiral fabrication technologies, chiral bio-interfaces have provided a new choice for diagnosing and treating many diseases and showed great potential for transforming medicine and clinical applications. To clarify the merits of chiral bio-interface in biomedical applications, we have collected and summarized current progresses into three aspects, drug delivery, antibacterial, antivirus and antitumor, and bio-maker detection.

### 5.1. Chiral Effects on Drug Delivery

In order to extend the half-life of drugs, promote drug absorption and increase drug targeting, new drug delivery systems are widely developed. The potential applications of chiral porous materials in the field of enantiomer separation are of great concern. Yang’s group synthesized a range of chiral mesoporous silica materials with chiral drugs [51]. The chiral drugs metoprolol R- and S-enantiomers adsorbed on chiral mesoporous silica materials were observed to exhibit significantly different release behaviors.

Recently, Kehr et al. utilized chiral surface functionalized mesoporous organic silica as a stimulus-responsive local drug delivery system [52]. They found the acidic pH environment stimulates the Hst/DOXPMO-NH_2_ and Hst/DOXPMO-PSS/PLL/PDL systems to deliver more drug molecules to cells. This property allows the pH-responsive self-assembled monolayers of Hst/DOXPMO-NH_2_ and Hst/DOXPMO-PSS/PLL/PDL for topical drug delivery to deliver higher doses of the drug molecule to cancer cells while reducing toxic side effects of the drug to normal tissues.

Next, Fan et al. prepared on-off-D-chiral mesoporous silica nanoparticles (CMSN) with chiral functional groups [53]. Achiral drug indomethacin (IMC) loaded On-Off-L-CMSN has a better anti-inflammatory effect than IMC-loaded On-Off-D-CMSN. This work provides valuable guidance for chiral materials in drug delivery and release [54].

### 5.2. Chiral Effects on Antibacterial, Antivirus and Antitumor

Biological pollution is a major problem in public health and biomedical research. Anti-adhesion is the first priority to overcome biological contamination. Wang et al. developed chiral anti-microbially adhered biomaterials through polymer surface stereochemistry [55]. The l-type showed stronger resistance to *E. coli* cells than the D-type. The preparation of polymer films using chiral differences is an advanced antimicrobial adhesion strategy.

With the rapid development of nanotechnology, a large number of papers have reported on the application of nanomaterials in antibacterial and anti-infection. The Kuang group explored that L-Cys CdTe modified with polycationic nine peptides has biomimetic nanoenzyme activity [12]. PCNP-L-Cys CdTe under 405 nm right-circular polarized light produced a large number of hydroxyl radicals and caused bacterial death. The chiral effect of nanomaterials provides a new avenue for the clinical treatment of bacterial infections.

Gold nanoparticles have been widely studied as antimicrobials with good biocompatibility, stability and low cytotoxicity. Functional gold NPs (D/L-Au NPs) with stable antimicrobial effects were synthesized with cysteine as the ligand [56]. The fatality of D-Au NP (96.8%) to *E. coli* is respective higher than L-Au NPs (65.4%), and higher than free D-cys, L-cys. This study provides a new option for designing effective chiral nano-antimicrobials for the treatment of bacterial infections. Similarly, Au-NBP is modified with D-glutamic acid (D-Glu) or L-glutamic acid (L-Glu) to form chiral Au-NBP [57]. Chiral glutamic acid can enhance the binding of Au-NBP to bacterial cell walls.

Photothermal therapy is a new effective bactericidal method and the combination of chiral nanomaterials will greatly improve the antibacterial performance. Sun et al. synthesized dipeptide-mediated chiral gold nanomaterials (D/L-GBP) and found that D-GBPs can effectively kill more bacteria than L-GBPs in vitro [58]. Mechanical analysis showed that the binding affinity of D-GBPs for *Staphylococcus aureus* protein A was higher than L-GBPs. D-type NP induced a stronger antimicrobial response than L-type NP. This research provides a promising tool for hand-shaped nanomaterials to fight bacterial infections.

The effect of molecular chiral antibacterial activity has been widely studied, but the effect of supramolecular chirality on bacteriostatic activity is still insufficient. The helical structure is one of the fundamental features of the extracellular matrix, so it is necessary to study supramolecular chiral-induced antibacterial activity. The Feng group synthesized four supramolecular chiral hydrogel agents by reacting gel factor molecules (LPF/DPF) with 2-amino-5-methylthiazole (MTZ) and 5-amino-1,3,4-thiadiazole-2-thiazole-2-thiazol (TDZ) [59]. The antibacterial activity of the D-MTZ and D-TDZ hydrogels was significantly higher than that of L-MTZ and L-TDZ. This study shows that the amplification effect of supramolecular chirality has a more significant effect on antibacterial activity than molecular chirality, which provides interesting insights into chiral antibacterial.

**Figure 9:**
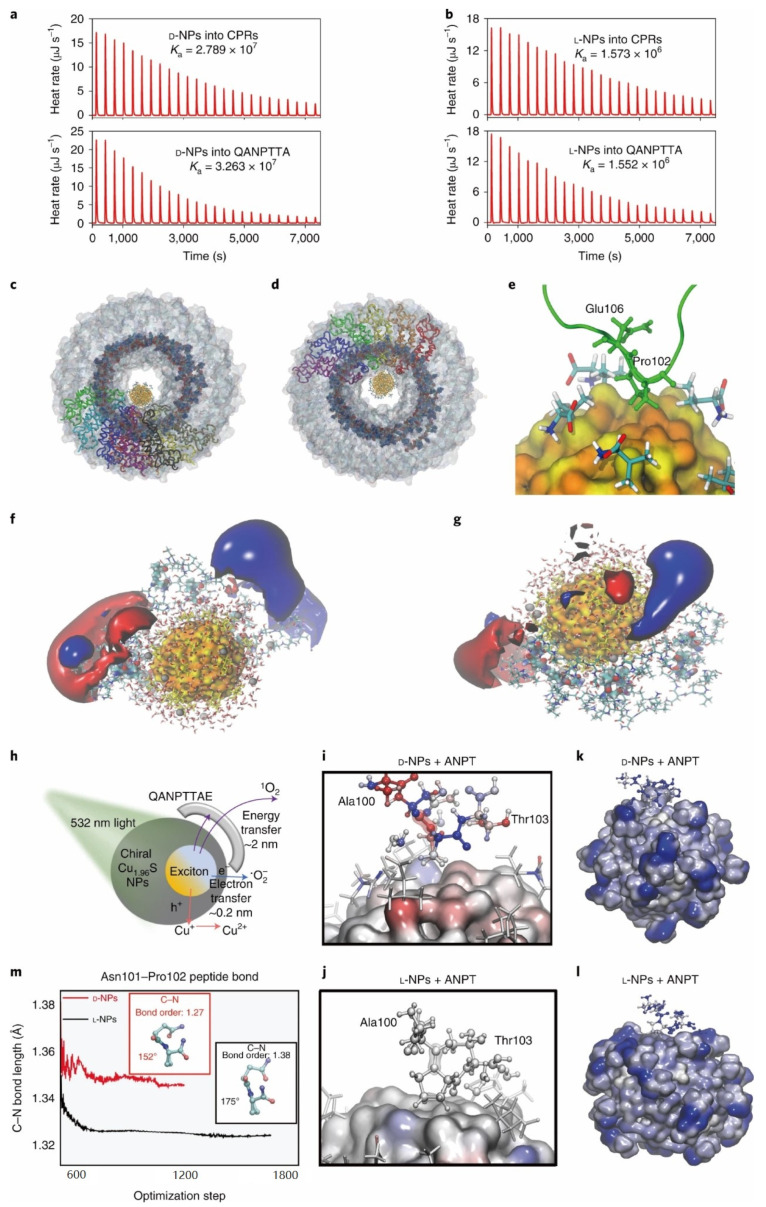
(**a**,**b**) Isothermal calorimetry data for CPRs and QANPTTA forming host-guest complexes with D-NPs and L-NPs. (**c**,**d**) MD simulation of the host–guest complex for CPRs with D-NPs and L-NPs. (**e**) Atomistic details of interaction between D-NPs and CP. (**f**,**g**) Isosurfaces for average electrostatic polarizations of D-NPs and L-NPs interacting with multiple QANPTTA segments of CP monomer in TMV capsid being illuminated. (**h**) Excitation, energy and charge-transfer processes between chiral CuS NPs and surrounding chemical structures. (**i**,**j**) Polarization maps for the Ala 100-Asn 101-Pro 102-Thr 103 fragment (ANPT) interacting with D-NP or L-NP. (**k**) Molecular organization of the D-NP-peptide complex for the ANPT fragment. (**l**) Molecular organization of the L-NP-peptide complex for the ANPT fragment. (**m**) Bond length for the Asn 101-Pro 102 peptide bond during the DFT geometry optimization for D-NP and L-NP interacting with the closest ANPT fragment [13].

Xu et al. found that chiral Cu_1.96_S nanoparticles can site-selectively cleave capsid in tobacco mosaic virus under sunlight. With D-penicillamine as surface ligands, the nanoparticles display high affinity to the capsid via a network of supramolecular bonds. They demonstrated that Pen-ligand-functionalized chiral Cu_1.96_S NPs could serve as effective photoactivated antivirals for plants and established a detailed mechanism of their virucidal action (Figure 9). These findings showed that nanoparticles combining site selectivity due to nanoscale chirality can be used as effective antiviral agents and revealed important aspects of light-matter interaction between chiral NPs and biomolecules [13].

Chen et al. have prepared a chiral black phosphorus nanosheet (BPNS) based on cysteine (Cys) surface engineering design. Compared to L-Cys-BPNS, D-Cys-BPNS exhibits approximately three times the cytotoxic effect on tumor cells, exhibiting chirality-dependent therapeutic behavior in vitro and in vivo. Chiral engineering is expected to open up new avenues and promote the application of BPNS in tumor phototherapy [14].

### 5.3. Chiral Effects on Biomarker Detection

**Figure 10:**
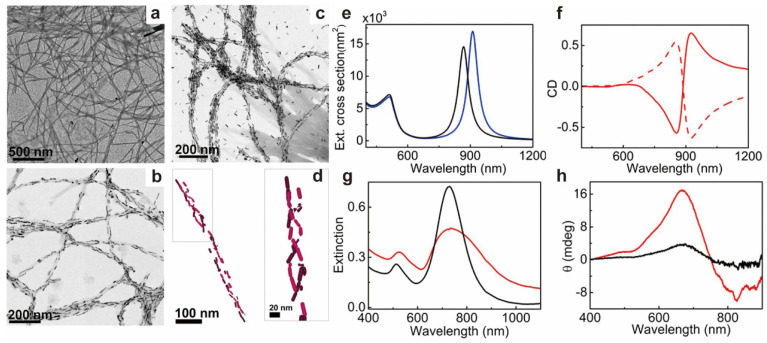
(**a**) TEM images of α-synuclein fibrils. (**b**,**c**) TEM images of Au NRs in the presence of α-synuclein fibrils for varying NR concentrations. (**d**) Cryo-TEM tomography reconstruction image of a composite fiber showing the 3D chiral arrangement of Au NRs. (**e**) Extinction spectra for an Au NR left-handed helical assembly calculated under the excitation of left (blue trace) and right-handed (black trace) circularly polarized, axially impinging plane waves. (**f**) Simulated CD spectra for normal incidence for right (solid curve) and left-handed (dashed curve) double helices of Au NRs. (**g**) Extinction and (**h**) CD spectra of Au NRs monitored after the addition of purified brain homogenates from healthy (black traces) and PD-affected (red traces) patients [62].

In recent decades, the application of biosensors and nanoprobes has become a hot research topic in chiral bioscience. A large number of chiral NPs have been prepared to study the dependence and recognition characteristics of biological systems on chiral molecules. It is found that its mechanism and sensitivity mainly depend on the specific interaction between NPs and ions or biomolecules (including DNA, RNA, protein, protein secondary structural elements and peptides). Especially in a physiological environment, a protein with high abundance tends to interact with chiral NPs. Researchers have begun to pay attention to the early detection of neurodegenerative diseases since 2018. Many important works show that chiral NPs, as a key pathogenic protein, especially a high molecular weight fibrillation protein, have potential application prospects in the diagnosis of neurodegenerative diseases. For example, the binding affinity of D-Fe_x_Cu_y_Se [60] and D-GSH-Au [61] to Aβ42 monomer is higher than that of L-NPs, and after further CPL irradiation, these two D-NPs are also better than L-NPs in inhibiting Aβ42 monomer aggregation and promoting Aβ42 fibril decomposition. In treating Parkinson’s disease, gold plasma nanorods can be used to detect the formation of amyloid fibrils based on a-synuclein (Figure 10) [62].

Due to their unique size effect and optical characteristics, metal and semiconductor nanoparticles have also developed rapidly in detecting many substances related to biomedical applications, such as bioengineering, biochemical indicators, pharmaceutical industry and food safety in different organisms [63]. Kim’s group described the D- and L-Pen modified AuNPs (Pen-AuNPs) based electrochemical sensors for enantiomerically selective identification of 3,4-dihydroxyphenylalanine (DOPA) [64]. As the AuNP size decreases, the oxidation peak potential of D/L-DOPA on L/D-Pen AuNP shifts more toward negative voltage. This phenomenon indicates that electrodes with smaller sizes of Pen-AuNP have better enantiomeric selectivity for D- and L-DOPA.

DNA carries the genetic information necessary for the synthesis of RNA and proteins, which is essential for the development and proper functioning of organisms. Xia et al. designed a stem-loop structure probe (molecular beacon) for detecting DNA with chiral molecularly modified electrochemical DNA (E-DNA) sensor [65]. They found that higher DNA molecules could be adsorbed on the L surface than on the D surface and the peak current on the L surface is greater than on the D surface, which can be used to improve the sensitivity and limits of detection.

Mandelic acid is a drug intermediate and fine chemical product with a wide range of uses. Water-soluble chitosan derivative Hydroxypropyl chitosan (HPCS) is prepared by amino protection using benzaldehyde to selectively introduce hydroxypropyl. HPCS constructs multi-walled carbon nanotubes (MWCNTs)-HPCS electrochemical sensors by combining covalent bonds with carboxyl group, and stereoselectively identifies mandelic acid enantiomers by cyclic voltammetry [66]. In addition, HPCS as a chiral selector Phenylalanine (Phe) and Trp enantiomer chiral differentiation, both showed chiral differences. This research opens up a new avenue for chiral interfaces for electrochemical identification.

Amino acids are the basic units that make up proteins and are widely used in the medical, food, and cosmetics industries. Chiral testing of amino acids is essential for life sciences and biomedicine. Li et al. obtained NALC-modified Au NPs by adsorbing N-acetyl-L-cysteine (NALC) with chiral structures onto the surface of Au NPs [67]. When L-tyrosine is added, the color change from red to purple can be clearly observed. However, after the uniform addition of D-Tyr, no color change occurs. In the ultraviolet-visible absorption spectrum, L-Thanh has a decrease in absorbance at 522 nm and a new absorbance peak at 630 nm, while D-Tyr has not changed significantly. These results suggest that NALC-Au NP enables selective identification and enrichment of Tyr enantiomers, which provides potential applications for the isolation of amino acid enantiomers.

Xu et al. explored the ability of chiral D/L cobalt hydroxide nanoparticles to detect reactive oxygen species in tumor-bearing mice based on magnetic resonance imaging and dual fluorescence signals. They designed an “On-Off” fluorescence probe. N-acetyl-l-cysteine and lipopolysaccharide were used to down-regulate and up-regulate the level of reactive oxygen species in tumors in tumor-bearing mice. The results of fluorescence imaging and magnetic resonance imaging showed that chiral cobalt hydroxide nanoparticles could effectively and dynamically monitor the level of reactive oxygen species in tumors, and the fluorescence or magnetic resonance imaging signals of D-type nanoparticles were better than those of L-type nanoparticles. These indicate that chiral nanoparticles with multiple optical and magnetic properties can be used for ultra-sensitive detection of reactive oxygen species in living cells and in vivo.

## 6. Conclusions and Outlook

From the viewpoint of fundamental research, the reported works showed different chiral effects with a natural or manmade chiral interface, and some of the results clearly contradict each other. It means a clear biophysical model is extremely required to be developed to clarify the chiral interactions at the molecule or assembly level. In addition, researchers should pay more attention to the underlying mechanism of chiral interactions with biological systems in detail, for example, chirality can induce gene expression or signal pathway changes involved in cell adhesion, migration, proliferation, differentiation and cell apoptosis [68]. Understanding the chiral effect on epigenetic patterns in different biological systems will help us better control biological fate. Noteworthily, the current research focuses on the surface chirality of two-dimensional substrates. The integration of molecule chirality and topographical chirality together on the three-dimensional bio-interfaces is meaningful for better mimicking in vivo cell microenvironments to study the cell fate, tissue repair and organism immune response.

From the aspect of application studies, the development of practical bio-material or bio-device that combines the excellent properties of chiral interface and biological effect will keep attracting attention. In addition, the interaction between chiral surface and biomarker plays a vital role in disease diagnosis or bio-detection. In the future, researchers will explore how to integrate the unique properties of materials and chiral moiety to realize the ideal performance in chiral-material-based therapeutics. As for biomedical application, the chiral bio-interface provides us a platform to study biomedical applications, such as drug delivery, antibacterial and antiviral activities, and biological signal detection. Chirality could enhance drug delivery systems, tumor markers, and biosensors, among other biomaterial-based technologies [69].

In summary, we reviewed the recent progress of chirality as a regulator for controlling cell fate, tissue repair, and organism immune response as well as the biological applications of chirality. Although recent research in this scientific field is still not detailed enough, and existing research cannot support our comprehensive and systematic explanation of the regulatory relationship between chiral interfaces and biological effects, some significant progress has been achieved in using chiral features to control cells or organisms. There are crucial questions and challenges for the current and further directions of chiral-biology interfaces that must and will be addressed in the future. We believe the chiral-biological interface is destined to play a huge role in the biological field.

## Data Availability

Not applicable.
